# Formulation and In-Vitro Characterization of Chitosan-Nanoparticles Loaded with the Iron Chelator Deferoxamine Mesylate (DFO)

**DOI:** 10.3390/pharmaceutics12030238

**Published:** 2020-03-07

**Authors:** Maria Lazaridou, Evi Christodoulou, Maria Nerantzaki, Margaritis Kostoglou, Dimitra A. Lambropoulou, Angeliki Katsarou, Kostas Pantopoulos, Dimitrios N. Bikiaris

**Affiliations:** 1 Laboratory of Polymer Chemistry and Technology, Chemistry Department, Aristotle University of Thessaloniki, 54124 Thessaloniki, Greece; marlazach@chem.auth.gr (M.L.); evicius@gmail.com (E.C.); marinera002@msn.com (M.N.); 2 Laboratory of General and Inorganic Chemical Technology, Department of Chemistry, Aristotle University of Thessaloniki, GR-541 24 Thessaloniki, Greece; kostoglu@chem.auth.gr; 3 Laboratory of Environmental Pollution Control, Department of Chemistry, Aristotle University of Thessaloniki, GR-541 24 Thessaloniki, Greece; dlambro@chem.auth.gr; 4 Lady Davis Institute for Medical Research and Department of Medicine, McGill University, Montreal, QC H3T 1E2, Canada; ageliki.katsarou@mail.mcgill.ca

**Keywords:** chitosan, nanoparticles, ionic gelation, tripolyphosphate, deferoxamine mesylate, iron chelator, nanoencapsulation, drug release

## Abstract

The objective of this study was to develop chitosan (CS) nanoparticles (NPs) loaded with deferoxamine mesylate (DFO) for slow release of this iron-chelating drug. Drug nanoencapsulation was performed via ionic gelation of chitosan using sodium tripolyphosphate (TPP) as cross-linker. Nanoparticles with a size ranging between 150 and 400 nm were prepared for neat CS/TPP with a 2/1 molar ratio while their yield was directly dependent on the applied stirring rate during the preparation process. DFO at different content (20, 45 and 75 wt %) was encapsulated into these nanoparticles. We found that drug loading correlates with increasing DFO content while the entrapment efficiency has an opposite behavior due to the high solubility of DFO. Hydrogen-bonding between amino and hydroxyl groups of DFO with reactive groups of CS were detected using FT-IR spectroscopy while X-ray diffraction revealed that DFO was entrapped in amorphous form in the CS nanoparticles. DFO release is directly dependent on the content of loaded drug, while model analysis revealed that the release mechanism of DFO for the CS/TPP nanoparticles is by diffusion. Treatment of murine RAW 264.7 macrophages with nanoencapsulated DFO promoted an increased expression of transferrin receptor 1 (TfR1) mRNA, a typical homeostatic response to iron deficiency. These data provide preliminary evidence for release of pharmacologically active DFO from the chitosan nanoparticles.

## 1. Introduction

Due to more than 40-year long clinical experience and low cost, the iron chelator deferoxamine (DFO) remains a first line therapeutic option to reduce iron burden in transfusion-dependent thalassemia patients [1]. The most significant challenge that DFO faces is its inability to be delivered effectively, since it is poorly absorbed in the gastrointestinal tract and has a short plasma half-life of approximately 20 min. In order to reach effective pharmacological concentrations, the drug is administered parenterally with the aid of a portable infusion pump at least 4–5 days per week and for 8–10 h each time. This rather strenuous procedure reduces compliance and compromises of quality of life.

Nanocarriers, such as biocompatible polymers, offer unique possibilities to overcome cellular barriers and improve drug delivery [2]. Their small sizes allow high diffusivity across membranes improving drug permeability. Nanoparticles (NPs) have a diameter of less than 200 nm and this can facilitate their cellular uptake via receptor-mediated endocytosis, fluid phase endocytosis or even passive diffusion; moreover the small size reduces their clearance by the mononuclear phagocyte system, which in turn increases their circulation time in blood [3,4,5,6]. Thus, the encapsulation of DFO in biocompatible nanoparticles aiming in a slower and more controlled release of the drug in the blood could provide an alternative means for drug administration, bypassing the compliance issue and improving quality of life. Released DFO should maintain its pharmacological properties, efficacy and safety profile.

Chitosan (CS) NPs have attracted a lot of attention as drug delivery vehicles due to their ability to protect the drug from degradation, to achieve higher drug-loading and to release it in a slow and sustained manner [7,8,9,10]. Furthermore, due to the hydrophilic properties of chitosan, CS NPs are suitable carriers for hydrophilic drugs, like DFO. Lately, CS NPs have shown promising results in delivering drugs for treatment of diabetes and cancer [11]. In another case, CS NPs were inoculated in the rabbit ocular surface and were able to interact and remain associated with the ocular mucosa for extended periods of time without causing inflammation [12]. A major challenge remains the size-controlled synthesis of CS NPs [13].

CS-tripolyphosphate (TPP) NPs are intended for in vivo administration because of their excellent biosafety profile. The ionic gelation method used for their preparation does not require the addition of any organic solvents, a fact that avoids the problem of elimination of residues prior to delivery into living organisms [14]. However, the reaction time, the stirring rate and the amount of tripolyphosphate added in the chitosan solution are factors requiring optimization [15,16,17,18,19].

The aim of the present work was to prepare CS nanoparticles loaded with the iron chelator DFO. We reasoned that since DFO exhibits a high water solubility and shares amino and hydroxyl groups with CS, it could be encapsulated in high amounts within nanoparticles. After optimizing mechanical stirring on the particle size during the ionic gelation proccess, we prepared CS NPs and characterized them as appropriate DFO drug carriers by several techniques such as scanning electron microscopy (SEM), Fourier transfrom infrared spectroscopy (FT-IR) and X-ray diffraction (XRD). We provide preliminary evidence that encapsulated DFO can be released from CS/TPP NPs and generate an iron-deficient phenotype in cultured cells.

## 2. Experimental

### 2.1. Materials

**Scheme 1:**
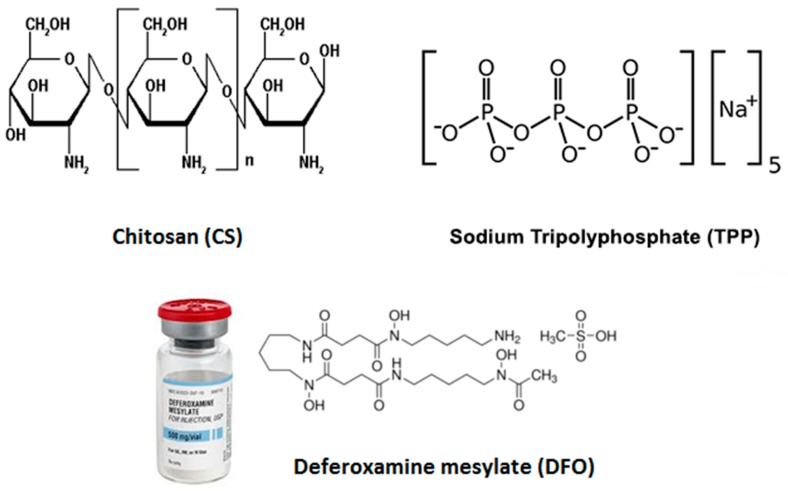
Chitosan, tripolyphosphate (TPP) and desferoxamine mesylate used for nanoencapsulation.

Chitosan of high molecular weight (MW: 350,000 g/mol, deacetylation degree >75% and viscosity 800–2000 cps), sodium tripolyphosphate (TPP) and ferric ammonium citrate were all purchased by Sigma-Aldrich Co (St. Louis, MO, USA). Deferoxamine mesylate was supplied from Mayne Pharma Inc. (Montreal Canada; Scheme 1). All other materials and solvents used in this study were of analytical grade.

### 2.2. Preparation of CS Nanoparticles

CS nanoparticles were prepared according to the ionotropic gelation method [20]. Blank nanoparticles were obtained upon the addition of TPP aqueous solution as an ionic crosslinking agent (final concentrations 1 mg/mL, respectively) to a CS acetic acid solution. CS/TPP ratio was 2/1 w/w and different stirring rates (400, 800 and 1200 rpm) were applied. For the preparation of drug-loaded nanoparticles, deferoxamine mesylate (DFO) aqueous solutions with CS were prepared and TPP was added dropwise in order to prepare the nanoparticles. Every sample was prepared in triplicate and the results represent the average value. Non-entrapped drug and disolved CS was removed by ultra centrifugation at 13,000 rpm for 20 min and reconstituted from the precipitate in fresh water (twice). The resulting suspension was frozen and lyophilized by a freeze-drier system (Scanvac, Coolsafe 110-4 Pro, Labogen Scandinavia) for 24 h at −108 °C to obtain a dried nanoparticle product. 

### 2.3. Characterization of CS Nanoparticles

#### 2.3.1. Morphological Characterization of Nanoparticles

The morphology of the prepared nanoparticles was studied using scanning electron microscopy (SEM; JEOL JMS-840 apparatus). The samples were covered with a carbon coating in order to provide good conductivity of the electron beam. Operating conditions were: accelerating voltage 20 kV, probe current 45 nA and counting time 60 s.

#### 2.3.2. Size Measurements of Nanoparticles

The intensity average particle size distribution of CS/drug nanoparticles was determined by dynamic light scattering (DLS) using a Zetasizer Nano Instrument (Malvern Instruments, NanoZS, ZEN3600, UK) operating with a 532 nm laser. A suitable amount of nanoparticles was dispersed in distilled water creating a total concentration 1% and was kept at 37 °C under agitation at 100 rpm. 

#### 2.3.3. Wide Angle X-ray Diffractometry (WAXD)

A wide angle X-ray diffractometry (WAXD) study of neat and drug encapsulated nanoparticles was performed over the range 2θ from 5 to 50°, at steps of 0.05° and counting time of 5 s, using a MiniFlex II XRD system from Rigaku Co. with Cu Ka radiation (λ = 0.154 nm).

#### 2.3.4. Fourier Transformation-Infrared Spectroscopy (FT-IR)

FT-IR spectra were obtained using a PerkinElmer FTIR spectrometer, model Spectrum 1000. In order to collect the spectra, a small quantity of freeze-dried nanoparticles was mixed with KBr (1 wt % nanoparticles) and compressed to form tablets. The IR spectra of these tablets, in absorbance mode, were obtained in the spectral region of 450–4000 cm^−1^ using a resolution of 4 cm^−1^ and 64 co-added scans.

#### 2.3.5. Differential Scanning Calorimeter (DSC)

A differential scanning calorimeter (DSC) study was performed using a Perkin–Elmer, Pyris Diamond DSC, calibrated with indium and zinc standards. Samples of 10.0 ± 0.1 mg sealed in aluminum pans were heated from room temperature until 200 °C at a heating rate of 20 °C/min under nitrogen atmosphere and held at that temperature for 5 min. Then the samples were supercooled to 30 °C at a cooling rate of 200 °C/min, held at this temperature for 5 min and subsequently heated again with 20 °C/min until 200 °C. 

#### 2.3.6. Evaluation of Drug Encapsulation and Release Rates

The drug content of nanoparticles was determined using a direct procedure. Lyophilized samples of nanoparticles (1 mg) were dissolved in a mixture of aqueous acetic acid solution (1% v/v)/ferric chloride (20 mg/mL) 50%/50% volume/volume and the solutions were assayed for drug content using a Shimadzu HPLC system with a CNW Technologies Athena C18 column (250 mm × 4.6 mm and 5 µm internal diameter) at 40 °C. Chromatographic station CSW was used for regression analysis and data acquisition. Concentration was determined by using an HPLC-UV apparatus at 430 nm as described in our previous study [21]. In brief, the HPLC system was operated isocratically, flow rate was set at 1.2 mL/min and injection volume was 20 μL. The mobile phase consisted of 27 mM KH_2_PO_4_/tetrahydrofurane/triethylamine (93:7:0.05 v/v/v). Each sample was measured in triplicate to obtain quite sharp peaks at retention time 6.6 min and chromatographic data were processed to concentration. Nanoparticle yield, drug loading and drug entrapment efficiency were calculated from Equations (1)–(3), respectively:

(1) $$\mathrm{Nanoparticles}\;\mathrm{yield}\;(\%)=\frac{\left(\mathrm{weight}\;\mathrm{of}\;\mathrm{nanoparticles}\right)}{\left(\mathrm{weight}\;\mathrm{of}\;\mathrm{polymer}\;\mathrm{and}\;\mathrm{drug}\;\mathrm{fed}\;\mathrm{initially}\right)}\;\times \;100$$

(2) $$\mathrm{Drug}\;\mathrm{loading}\;(\%)=\frac{\left(\mathrm{weight}\;\mathrm{of}\;\mathrm{drug}\;\mathrm{in}\;\mathrm{nanoparticles}\right)}{\left(\mathrm{weight}\;\mathrm{of}\;\mathrm{nanoparticles}\right)}\;\times \;100$$

(3) $$\mathrm{Entrapment}\;\mathrm{efficiency}\;(\%)=\frac{\left(\mathrm{weight}\;\mathrm{of}\;\mathrm{drug}\;\mathrm{in}\;\mathrm{nanoparticles}\right)}{\left(\mathrm{weight}\;\mathrm{of}\;\mathrm{initially}\;\mathrm{used}\;\mathrm{drug}\right)}\;\times \;100$$

The release rate of DFO from prepared nanoparticles was also determined using an HPLC UV apparatus at 430 nm, as described in our previous study [21].

#### 2.3.7. Cell Culture

Murine RAW 264.7 macrophages were grown in Dulbecco’s modified Eagle medium (DMEM) containing 10% fetal bovine serum, 100 U/mL penicillin and 100 mg/mL streptomycin (Wisent Inc., St-Bruno, QC).

#### 2.3.8. Quantitative Real-Time PCR (qPCR)

At the end points, the cells were scraped, harvested and washed in cold phosphate buffered saline (PBS). The cell pellets were then lysed in RLT Buffer. Total RNA was isolated from cell lysates using the Rneasy Mini kit (Qiagen). Purity was assessed by 260/280 nm absorbance ratios. qPCR was performed by using gene-specific primers (Table 1), as previously described [22] with ribosomal protein S18 (rS18) as a housekeeping gene for normalization. qPCR results are represented as fold changes compared to RAW 264.7 macrophages treated with PBS.

#### 2.3.9. Statistical Analysis

Quantitative data were expressed as mean ± standard error of the mean (SEM). Comparisons were made using the unpaired Student’s *t* test. A probability value *p* < 0.05 was considered to be statistically significant.

## 3. Results and Discussion

### 3.1. Size and Morphology of CS/TPP Nanoparticles

The particle size and size distribution of chitosan nanoparticles are important parameters for the development of physically and chemically stable nanocarriers, since these properties influence significantly their biological performance and drug release profile [22,23]. The effect of the CS/TPP ratio on the size of nanoparticles was studied in our previous work, where we obtained the lowest nanoparticle size at a CS/TPP w/w ratio near 2/1–4/1 [24,25]. The addition of higher TPP content caused rapid nanoparticles aggregation resulting in higher sizes [26].

However, along with the ratio between CS and TPP, the stirring conditions can also be crucial in determining what kind of particle shape and size dispersity is obtained due to different growth rates. The mechanical energy associated with the reaction stirring speed may exceed the electrostatic repulsion energy between nanoparticle positive surface charges, thus eventually triggering aggregation phenomena [27,28]. Thus, at the present work, the crucial role of the stirring speed was also evaluated, as being one of the factors, which is directly implicated in aggregation. The CS/TPP 2/1 w/w ratio was selected and different stirring speeds like 400, 800 and 1200 rpm were applied. Table 2 shows nanoparticle yields at different stirring rates as well as the prepared sizes.

**Figure 1:**
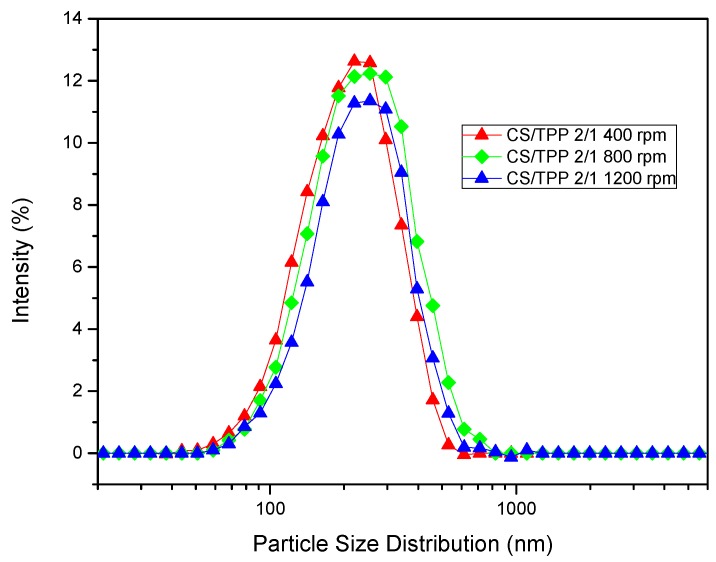
Dynamic light scattering (DLS) curves of CS/TPP 2/1 nanoparticles prepared at different stirring rates.

In agreement with previous studies [13], the highest percentage of nanoparticles was obtained at a stirring rate of 800 rpm (85.75%), even though the differences with the other speeds are not so high. At lower and higher stirring rates (400 and 1200 rpm) slightly lower nanoparticle yield was produced while particle size was not significantly affected (Table 2). The dispersion and particle distribution of nanoparticles as measured by DLS (Figure 1) was also similar in all stirring rates with very small and apparently statistically insignificant differences. These findings suggest that the stirring speed during ionic gelation affects the reaction yield, and therefore, by manipulating this parameter a greater proportion of nanoparticles of a given size range could be obtained.

**Figure 2:**
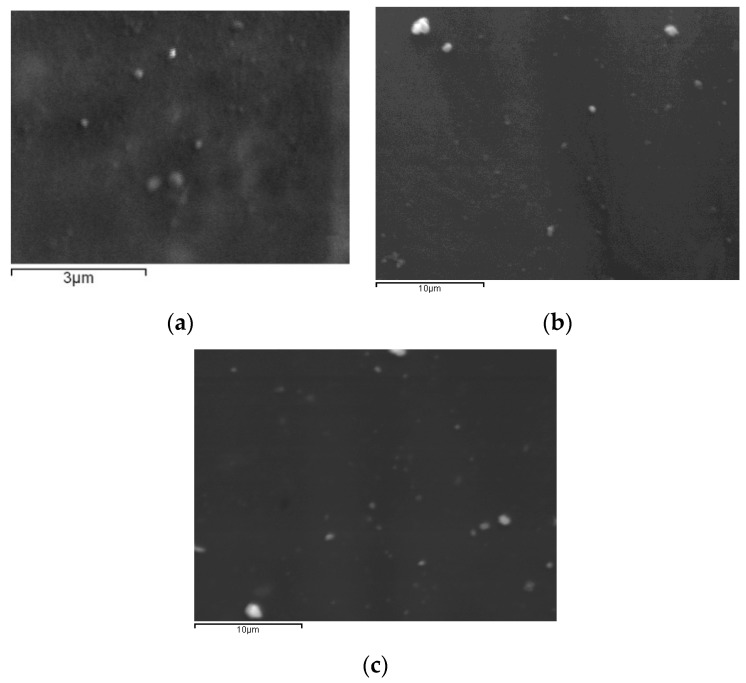
SEM micrographs of CS/TPP 2/1 nanoparticles of CS/TPP 2/1 nanoparticles prepared at different stirring rates: (**a**) 400 rpm, (**b**) 800 rpm and (**c**) 1200 rpm.

**Figure 3:**
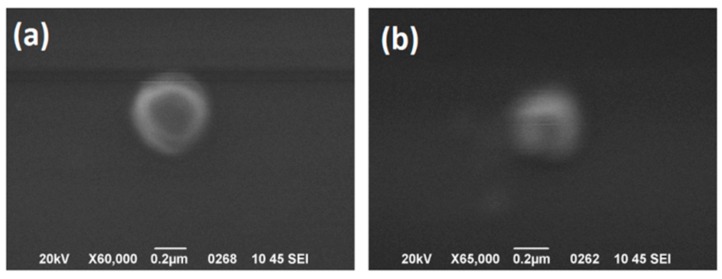
SEM micrographs of CS/TPP 2/1 nanoparticles prepared at different stirring rates and at higher magnification (0.2 μm): (**a**) 400 rpm and (**b**) 800 rpm.

The morphology of the nanoparticles was likewise not affected by variations in the stirring rates. Thus, the nanoparticle shape was essentially identical under all conditions (Figure 2) and as can be seen at higher magnification (Figure 3), in almost all cases spherical. The zeta potential due to amino groups of CS was in all nanoparticles positive and ranged between 37.6–40.1 mV. Thus, for DFO encapsulation, CS/TPP 2/1 w/w ratio was selected and the solution stirring speed was maintained at 800 rpm. 

### 3.2. CS/TPP Nanoparticles Loaded with DFO

DFO is very soluble and thus its loading into CS/TPP nanoparticles could be challenging. In order to evaluate this, we generated and studied the characteristics of CS nanoparticles with variable DFO content of 20, 45 and 75 wt %. As can be seen from Table 3 the particle size increased slightly by increasing DFO concentration. Measurements were performed with DLS at 25 °C and the data show a direct correlation between drug content and NP diameter. This is because DFO can interact via -OH and mainly via carbonyl groups with the CS amino groups forming an amide hydrogen bond. These interactions, in comparison also with reduced CS amount, could decrease CS’s availability for ionic interactions with TPP. Thus, increasing DFO concentrations lead to reduced TPP ionic interactions and thereby bigger NP size. This, in comparison with drug loading could affect DFO release. The DFO content also affects the other characteristics of CS nanoparticles; thus, the nanoparticle’s yield is progressively reduced by increasing DFO amount. This could be attributed to the low entrapment efficiency of DFO in nanoparticles, which is also reduced by increasing DFO content. As can be seen in all cases only a small percentage of DFO is loaded in nanoparticles (about 50 wt % in all conditions), which means that a big DFO amount was in solution. This is due to the high water solubility of DFO. 

**Figure 4:**
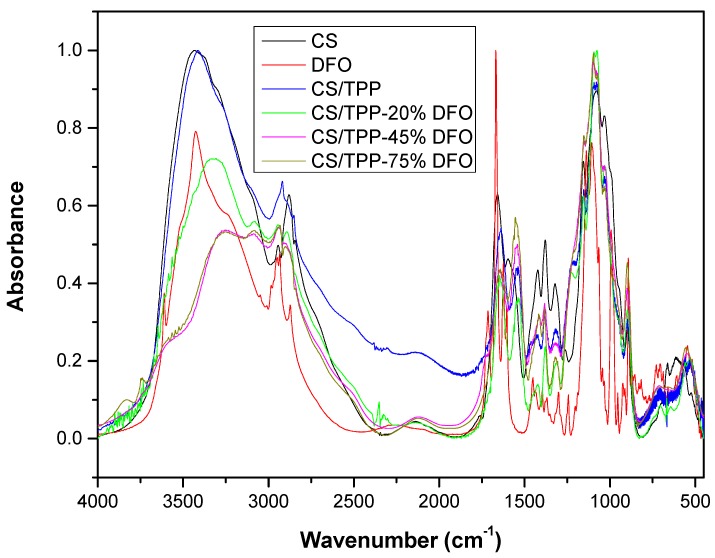
FTIR spectra of CS, DFO, CS/TPP and their nanoparticles containing different amounts of DFO.

It is known that drug release is largely dependent on the physical state of the compound that is encapsulated or dispersed in a polymeric matrix. It also counts on the extent of interactions with the polymeric matrix and particularly the ability to form hydrogen bonds. DFO contains hydroxyl, carbonyl and secondary amino groups, which could interact with the primary amino groups of the CS. To explore these possible reactions, FTIR spectra of all samples were recorded and are shown in Figure 4.

The broad peak around 3600–3000 cm^−1^ in neat CS should be assigned to the stretching vibration of –OH, the extension vibration of the >NH, and the intermolecular hydrogen bonds of the polysaccharide. Indeed, CS has a strong absorbance at 3428 cm^−1^ due to its –OH groups while primary amines have two peaks in this region (3280 and 3098 cm^−1^) due to NH_2_ groups and some lower intensity peaks (in the range 2921–2879 cm^−1^), which are attributed to stretching vibrations of methylene groups in the polymeric chain. There are weak absorption peaks at 1639 and 1545 cm^−1^ corresponding to amide I and amide II, respectively, which indicate that chitosan had a high deacetylation degree, while the absorption at 1075 cm^−1^ is due to asymmetric stress of the C–O–C bond. The absorbance at 1150 cm^−1^ is characteristic of asymmetric vibration of the b-(1-4) glycosidic bond [29,30]. DFO showed a small peak at 2310 cm^−1^ (free –OH groups), a sharp band at 3423 cm^−1^ due to –OH stretching vibrations and a broad peak (shoulder) at 3233 cm^−1^ that is assigned to N–H stretching vibrations.

**Figure 5:**
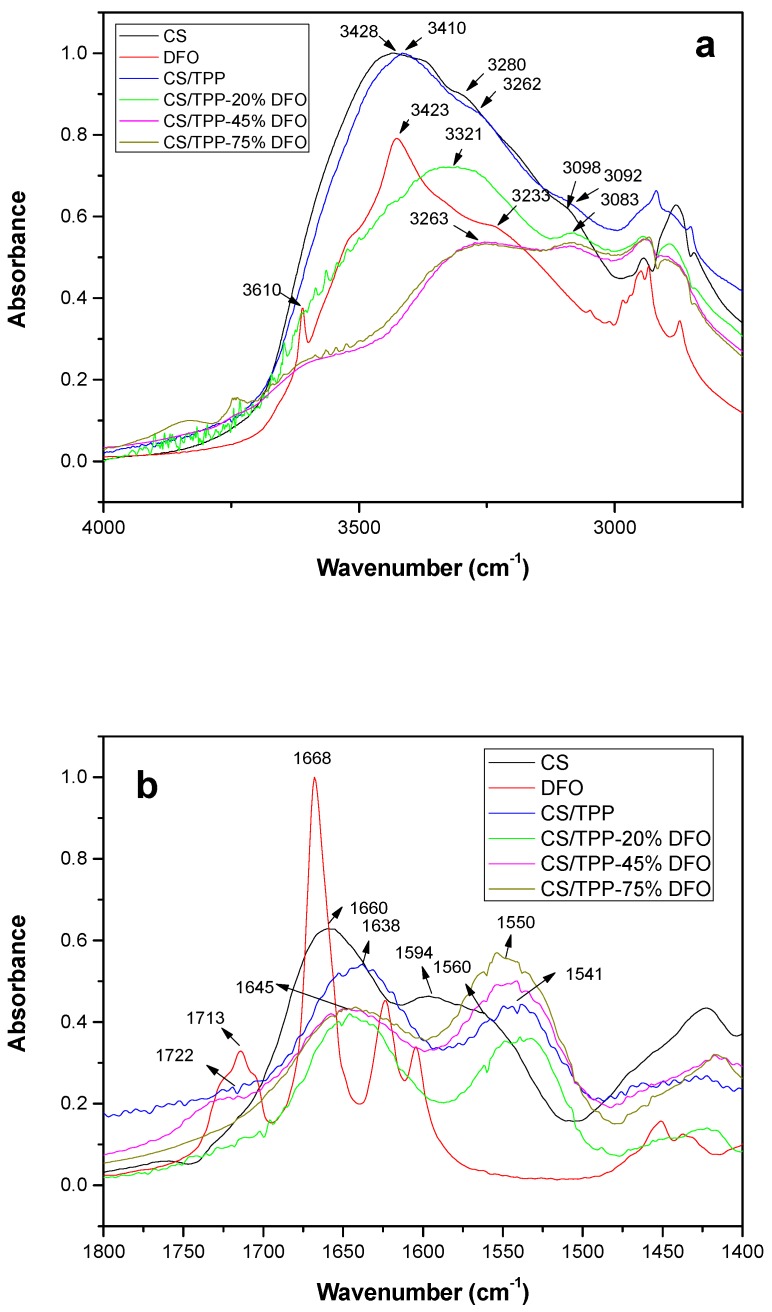
FTIR spectra of CS, DFO, CS/TPP and their nanoparticles containing different amounts of DFO in the areas of (**a**) of hydroxyl groups absorption and (**b**) carbonyl groups absorption.

The comparative spectra, in case of CS nanoparticles, are presented in Figure 5a,b in the areas of hydroxyl and carbonyl groups absorptions, respectively, where several shifts of the characteristic peaks can be clearly seen. Hydroxyl group bands in CS/TPP samples are recorded at 3410 cm^−1^ and amino stretching at 3262 and 3092 cm^−1^. These shifts are clearly due to the ionic interaction taking place between CS and TPP for the formation of nanoparticles. However, when DFO is incorporated the –OH band was shifted at 3321 cm^−1^, at CS/TPP with 20 wt % DFO without being separated from amino groups, while a small peak was recorded at 3083, due to the shift of –NH_2_ groups from 3092 cm^−1^. It also can be seen that at nanoparticles containing 45 and 75 wt % DFO, –OH peak was recorded at even lower wavenumbers (3263 cm^−1^). This shift was too high and is clearly due to the formation of hydrogen bonds between OH or amino groups of chitosan and DFO, since neat DFO had a peak at 3466 cm^−1^.

Figure 5b shows that neat CS had a broad band absorption at 1660 cm^−1^ and an amide carbonyl at 1545 cm^−1^ [31]. Both have been shifted to lower wavenumbers, 1638 and 1541 cm^−1^, respectively, when CS interacts with TPP. Neat DFO has also several peaks in these areas as in 1713, 1668 and two smaller intensity at lower wavenumbers. It is characteristic that in all CS/TPP/DFO nanoparticles these peaks were not recorded and it seemed that the high intensity peak at 1668 cm^−1^ was shifted to lower wavenumbers and merged with the carbonyl absorption of CS/TPP forming a new peak with absorption at 1645 cm^−1^. This finding indicates that the carbonyl peaks of DFO participate dalso in hydrogen bond interactions with CS.

**Figure 6:**
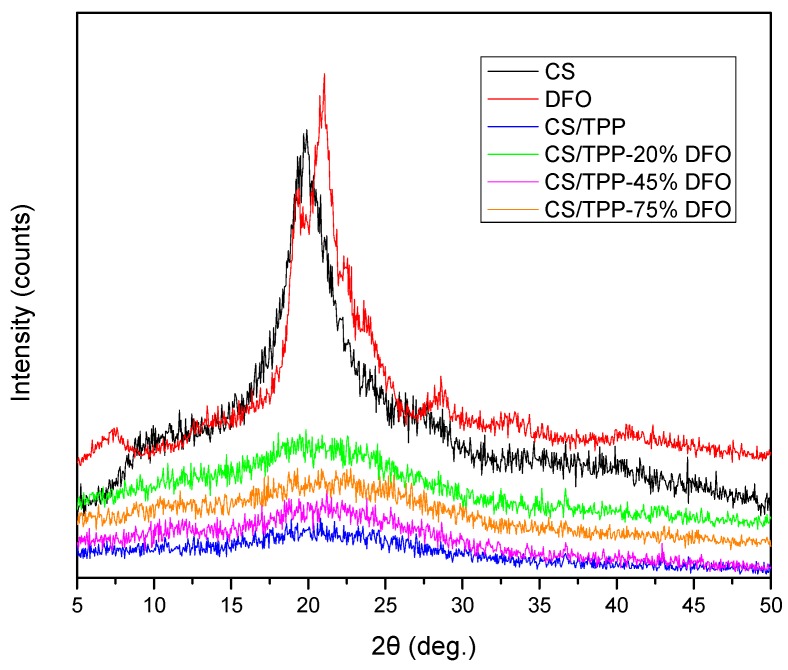
XRD patterns of CS, DFO, CS/TPP and their nanoparticles containing different amounts of DFO.

The physical state of the drug is also crucial for its release behavior and this was studied by XRD. As can be seen from Figure 6 neat CS exhibited the weak diffraction peaks centered at diffraction angle 2θ = 11.9°, and sharp diffraction peaks at 2θ = 20° were indicative of the high degree of crystallinity morphology of chitosan [32]. The addition of TPP had as a result to form an amorphous material (nanoparticles) since a hallow broad peak was recorded. So, it is clear that the ionic gelation and the evolved interactions between CS/TPP reduced the ability of CS chains to fold and crystallize. Concerning the CS/TPP nanoparticles with different amounts of DFO it can be seen that all these were also amorphous, while it is clear that DFO was a crystalline compound with many characteristic peaks at 2θ = 19.32°, 21.03°, 22.56°, 23.98° and 28.46°. In all XRD patterns of CS/TPP/DFO NPs only a broad peak at 2θ = 22° was recorded. The nanoparticles were characterized by the absence of any crystalline peak, which indicates that DFO was encapsulated (entrapped) in the amorphous form. This is probably due to the extensive hydrogen bonds formed between DFO and CS, as concluded by FT-IR spectroscopy [33].

**Figure 7:**
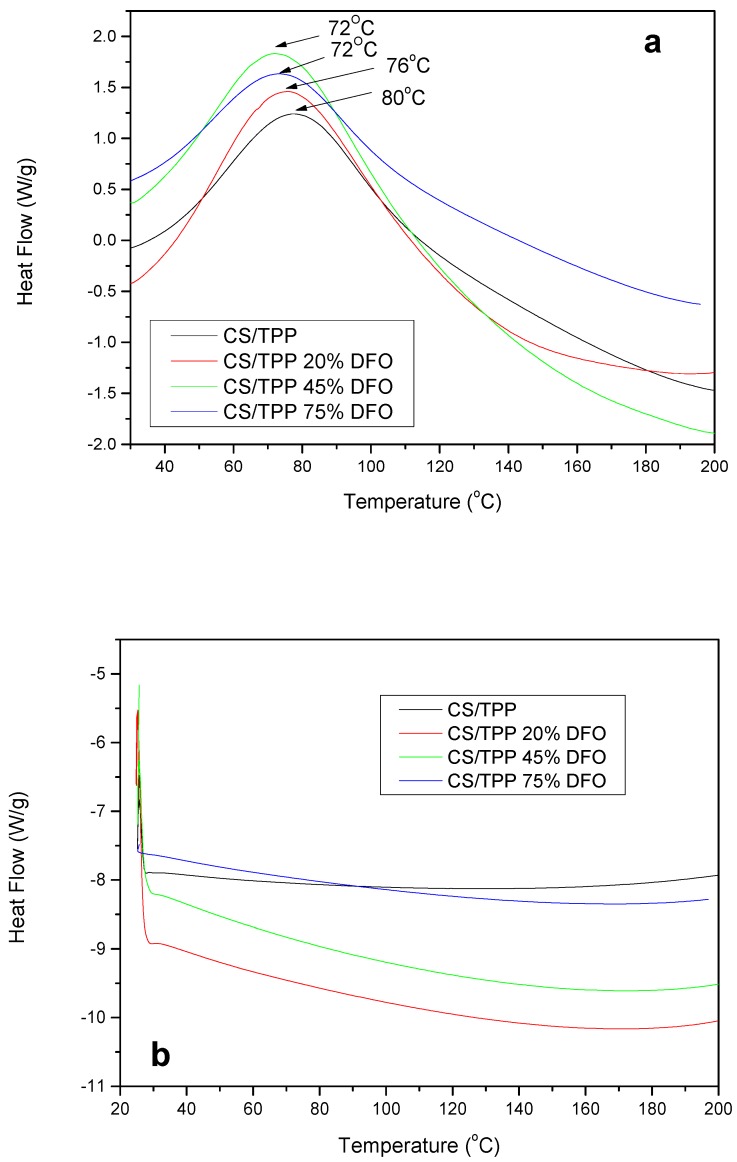
DSC thermograms of CS/TPP and their nanoparticles containing different amounts of DFO. (**a**) First heating and (**b**) second heating.

The drug amorphization was also verified by DSC. As can be seen in Figure 7a there was a broad peak recorded in CS/TPP due to dehydration of nanoparticles. CS is a very hydrophilic material and absorbed about 12–20 wt % of water at room temperature. This water was evaporated during heating of CS/TPP given a broad endothermic peak with a maximum about 80 °C. For this reason, no peak was recorded during the second scan after cooling from 200 °C (Figure 7b). A similar behavior was also observed for the CS/TPP nanoparticles encapsulated with DFO. All samples gave a broad peak during first heating and a line during second heating. The dehydration temperature seems to be dependent on DFO and as can be seen from Figure 7a was slightly reduced by increasing the DFO amount until 72 °C. The addition of DFO and the evolved interactions with CS reduced the available free groups and thus their ability to absorb more water, or the ability of absorbed water to be easily removed from these nanoparticles.

### 3.3. Drug Release from CS NPs

**Figure 8:**
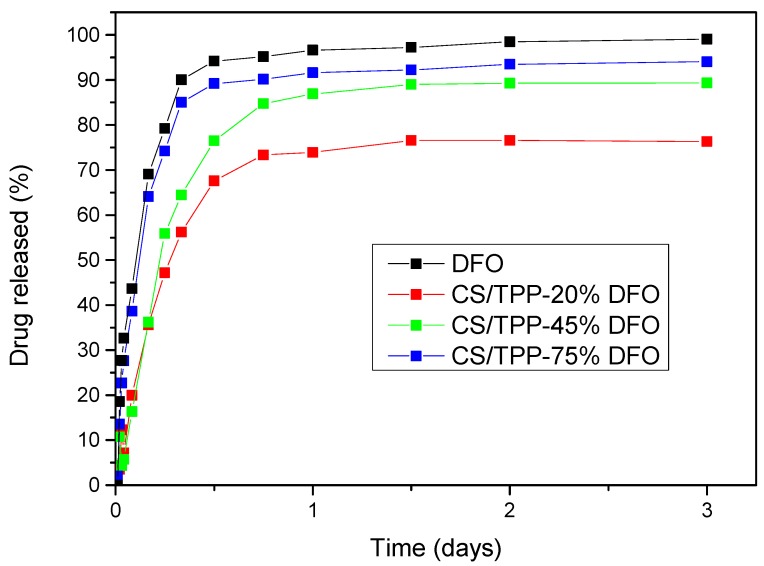
Kinetics of DFO release from CS nanoparticles (NPs) with variable DFO content.

DFO release from CS NPs is presented in Figure 8. It is clear that DFO release was mainly dependent on the amount of added DFO and at a lower extent on time, which is in good agreement with our previous study using poly(ε-caprolactone)-block-poly(propylene adipate) copolymers as appropriate matrices for nanoencapsulation of DFO [34]. A lag phase was observed in DFO release from CS NPs with lower drug content (20% and 45%) compared to free DFO, which is freely soluble. The lag phase was absent in CS NPs with 75% DFO and the release behavior was closer to that of neat DFO. This is because as was found from Table 3 a high percentage of DFO was loaded into nanoparticles (about 40%). These data suggest that increased DFO encapsulation improved its release profile from CS NPs. However, in all cases the release peaked at 1 day and then reached a plateau. Additionally, it seemed that in all cases and mainly in CS nanoparticles containing 20 and 45 wt % DFO the release was not complete and some amount remained in nanoparticles. This behavior could be attributed to the strong interactions that are taking place between DFO and CS, which was proved by FTIR, reducing the diffusion of DFO from nanoparticles.

Next, we attempted to model/analyze the experimental release data and to correlate them with the nanoparticles composition. Although at a first glance the release curves for pure drug and for CS/TPP nanoparticles appeared quite similar, it is noteworthy that they correspond to completely different physical mechanisms. In all release cases, taking into account that the characteristic release time is several hours and there is intense mixing, it can be assumed that mass transfer in bulk does not affect the release process.

The release mechanism for a pure drug is the drug dissolution. This process is usually described by a generalized Noyes–Whitney equation that takes into account finite rate dissolution kinetics [35]. The solution of this equation in the present experiment is a single exponential curve. The fitting of such a curve to the corresponding experimental data is of medium success. A possible reason for this is that the theory considers that the final bulk concentration is the equilibrium one (drug solubility). However, this is not clear in the present case since the final release of almost 100% implies that the final bulk concentration of the drug may not correspond to its solubility. In such a case the scheme of the release curve is strongly affected by the size distribution of the drug particles. In principle, this distribution can be reconstructed by the shape of the release curve. However, such a task is outside of the scope of the present study, which focuses on the nanoparticles release behavior.

**Figure 9:**
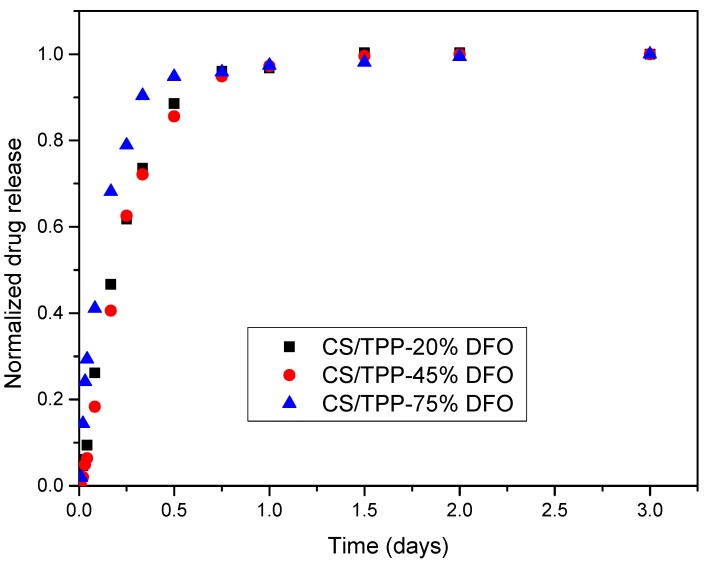
Normalized drug release data for three types of nanoparticles.

Examining the release from nanoparticles the first step is to isolate the limiting behavior from the dynamic one. The asymptotic release fraction for the 20, 45 and 75 DFO type of nanoparticles was 76.3%, 89.3% and 94%, respectively. It is clear that there was an amount of drug permanently bound to the CS/TPP matrix. This amount decreased as the drug content increased, leading to an increase of asymptotic released percentage. The next step is to construct the normalized release curves. It can be done by simply dividing the data with the corresponding asymptotic value. The normalized curves are shown in Figure 9 where the difference in release dynamics for the three drug contents is evident. These normalized curves have to be described by the appropriate model.

**Figure 10:**
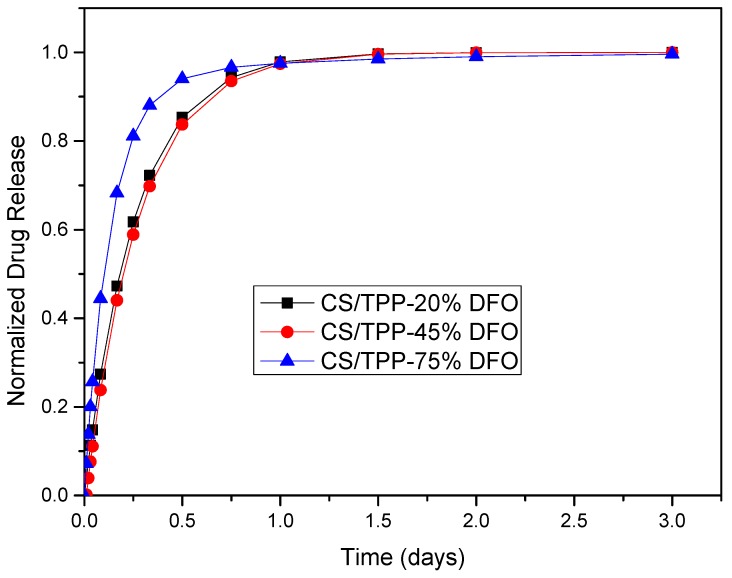
Comparison between experimental (symbols) and model (lines) normalized drug release results for three types of nanoparticles.

The release mechanism for the CS/TPP nanoparticles is a diffusive one. The DFO molecules diffuse through the CS/TPP structure until reaching the bulk fluid. There are several simple models in the drug release literature for the modeling of this diffusion process. A basic classification of these models is between the Fickian and non-Fickian type of diffusion [36]. These simple models typically refer to the initial stages of the release process. The present data are extended up to the completion of the release process; therefore, a different model is needed. In that case the process is described by the solution of the transient diffusion equation in the nanoparticle. This is a partial differential equation and in principle it must be solved numerically. However, in the special case of Fickian diffusion in a spherical particle an analytical solution in the form of infinite series exists [37]. An alternative approach is an approximate solution based on a polynomial representation known as linear driving force formula (LDF) [38]. This approach leads to an exponential form of the normalized release data:

(4) $$N_{R}=1-\exp(-\frac{15Dt}{R^{2}})$$

where N_R_ is the normalized released drug fraction, t is time, R is the nanoparticle radius and D is the effective diffusion coefficient of DFO in the CS/TPP matrix. The above equation is fitted to the experimental data shown in Figure 9. The value of R for each type of nanoparticle was calculated from the diameters appeared in Table 2. The fitting was very successful as it appeared in Figure 10 presenting comparison between experimental and modeling results. The LDF formula ignored the initial diffusion burst, which was included in the infinite series solution. The fact that LDF was enough to describe the present data implies that there was a lack of drug at the periphery of the particles. This was compatible to the observed release lag referred above.

The diffusion coefficients extracted from the fitting procedure are D = 0.684 × 10^−19^ m^2^/s, 1.43 × 10^−19^ m^2^/s and 3.65 × 10^−19^ m^2^/s for the three types of nanoparticles (20, 45 and 75 DFO) respectively. As it is expected the diffusion coefficient increased as the content of the drug in the particles increased. This increase appeared to be non-linear becoming more intense for larger drug contents. The data of diffusion coefficient were correlated with respect to the percentage drug content C in the nanoparticles (appearing in Table 2) through the fitted equation D = (0.0723C^2^ − 0.706C + 2.39) × 10^−19^ m^2^/s.

### 3.4. Evidence that DFO Released from CS NPs Operates as a Functional Iron Chelator in Cultured Cells

Cellular iron metabolism is post-transcriptionally regulated by the IRE/IRP system [39]. In iron deficient cells, “iron regulatory proteins” IRP1 and IRP2 bind to “iron-responsive elements” (IREs) in the untranslated regions of several mRNAs encoding proteins of the iron metabolism and control their expression. This homeostatic adaptation program promotes the iron uptake and prevents iron storage or export. Iron uptake is mediated by transferrin receptor 1 (TfR1), which is induced in iron-deficient cells upon stabilization of its mRNA by IRE/IRP interactions. Conversely, TfR1 is suppressed in iron-loaded cells due to inactivation of IRPs, which allows TfR1 mRNA degradation. Thus, expression of TfR1 mRNA is a common marker for cellular iron status.

**Figure 11:**
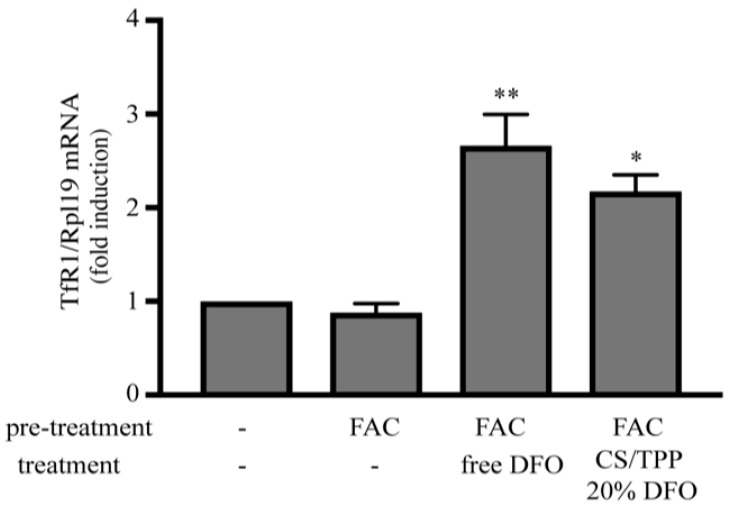
CS/TPP NP-encapsulated DFO promotes induction of TfR1 mRNA in RAW 264.7 macrophages.

We utilized murine RAW 264.7 macrophages as a cell culture model to address the iron-chelating properties of DFO released from CS NPs. The cells were either left untreated or pretreated with 30 μg/mL ferric ammonium citrate (FAC) for 24 h. Subsequently, the cells were washed and left untreated or treated with 100 μM free DFO or CS NP-encapsulated DFO for 18 h. The data in Figure 11 show that both free and CS NP-encapsulated DFO induced TfR1 mRNA levels, which is indicative of effective iron chelation. In preliminary experiments, we found that empty nanoparticles had no effects on TfR1 expression (not shown). Presumably, and based on the data in Figure 8, the release of DFO from the CS NPs was incomplete. Nevertheless, assuming a 70% release efficiency, the effective concentration of free DFO was well above 50 μM, which is known to trigger iron deficiency in cultured cells [40].

## 4. Conclusions

Favorable sizes of CS NPs were prepared appropriately for in vivo applications. Our data suggest an experimental optimum of CS/TPP ratio 2/1 w/w and of stirring rate 800 rpm for the highest nanoparticles yield. DFO, a drug widely used for the treatment of transfusion-dependent beta-thalassemia, was successfully encapsulated in these nanoparticles with high drug loading ranging from 11% to 40%, depending on the DFO amount. FT-IR spectroscopy identified strong interactions between the nanocarriers and the drug. These interactions may lead to the amorphization of DFO into nanocarriers, as was detected by XRD studies. Our experiments suggest that CS NP-encapsulated DFO was appropriately released and operated as an iron chelator in a cell culture model. Nevertheless, these preliminary data did not provide any clue on whether NP-encapsulated DFO is suitable for therapeutic applications, and further work with animal models is required to assess the pharmacological potential of this drug in vivo.

## Figures and Tables

**Table 1:** List of gene-specific primers used for qPCR.

Gene	GenBank Accession	Forward Primer Sequence	Reverse Primer Sequence
TfR1	NM_011638.4	AGCCAGATCAGCATTCTCTAACT	GCCTTCATGTTATTGTCGGCAT
Rpl19	NM_009078.2	AGGCATATGGGCATAGGGAAGAG	TTGACCTTCAGGTACAGGCTGTG

**Table 2:** Nanoparticle yield (%), particle size and zeta potential of CS/TPP 2/1 at different stirring rates.

^a^ Sample	Nanoparticle Yield (%)	Nanoparticle Size (nm)	Pdi	Zeta Potential (mV)
CS/TPP 2/1 400 rpm	77.50 ± 1.84	243 ± 15	0.62	38.45
CS/TPP 2/1 800 rpm	85.75 ± 1.12	287 ± 17	0.78	37.59
CS/TPP 2/1 1200 rpm	82.51 ± 0.93	269 ± 21	0.54	40.11

^a^ Three measurements per condition were obtained. Standard deviation (σ) has been calculated using the following formula: σ= [∑(x−mean)^2/N.

**Table 3:** Nanoparticle size, yield (%), drug loading (%) and entrapment efficiency (%) of CS/TPP/DFO at various DFO concentrations.

^a^ Sample	Nanoparticle Diameter (nm)	Nanoparticle Yield (%)	Drug Loading (%)	Entrapment Efficiency (%)
CS/TPP_blank	287 ± 17	85.75 ± 1.12	-	-
CS/TPP_20%DFO	304 ± 25	76.88 ± 1.12	11.55 ± 1.47	44.45 ± 1.39
CS/TPP_45%DFO	448 ± 31	61.46 ± 0.87	17.02 ± 2.12	26.15 ± 1.05
CS/TPP_75%DFO	528 ± 35	35.47 ± 1.34	40.09 ± 4.22	18.96 ± 0.89

^a^ Three measurements per condition were obtained. Standard deviation (σ) has been calculated using the following formula: σ= [∑(x−mean)^2/N.
